# Primary mediastinal leiomyoma: a case report

**DOI:** 10.4076/1757-1626-2-8555

**Published:** 2009-07-16

**Authors:** Yassine Ouadnouni, Abdellah Achir, Salma Bekarsabein, Mohammed Bouchikh, Mohammed Smahi, Yassine Msougar, Najat Mahassini, Abdellatif Benosman

**Affiliations:** 1 Department of Thoracic Surgery, Ibn Sina University HospitalRabatMorocco; 2 Department of Anatomical Pathology, Ibn Sina University HospitalRabatMorocco

## Abstract

Leiomyoma of the mediastinum is rare. We report a case of a 57-year-old woman with a cervical mass diving to the intrathoracic. Chest radiography and computed tomography revealed a mass in the right superior mediastinum. The tumor was enucleated by cervicotomy. Histologically, the tumor was diagnosed as leiomyoma.

## Introduction

Leiomyoma of the mediastinum is extremely rare. Only 12 cases of solitary leiomyoma that developed from small vessels in the soft tissue of the mediastinum wall have been reported in the English literature [1]. In the present paper, we will be discussing the case of a 57-year-old woman having a primary mediastinal leiomyoma treated by surgery.

## Case presentation

**Figure 1.**
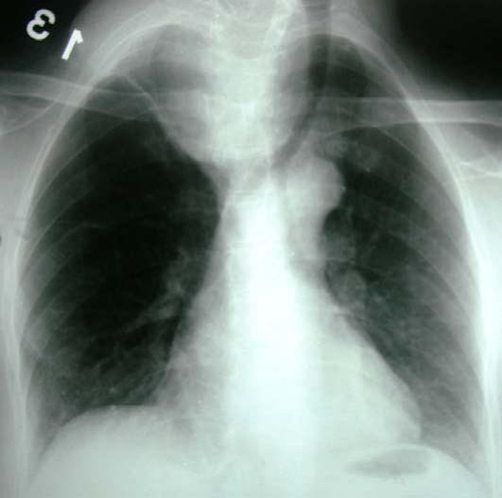
Chest radiography reveal a large soft tissue mass occupying the superior right hemithorax.

**Figure 2.**
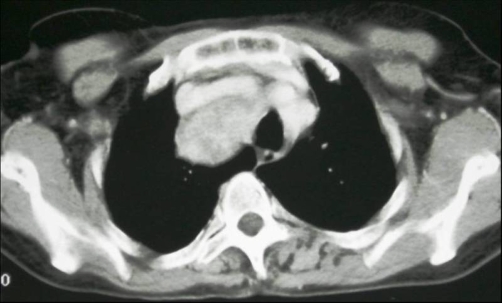
Computed tomography shows a well-circumscribed heterogeneous tumor in the right superior mediastinum.

**Figure 3.**
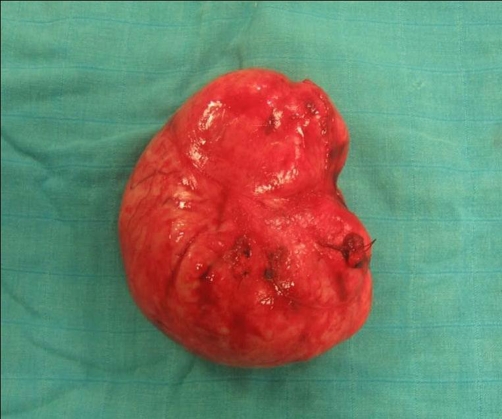
A large encapsulated tumor.

**Figure 4.**
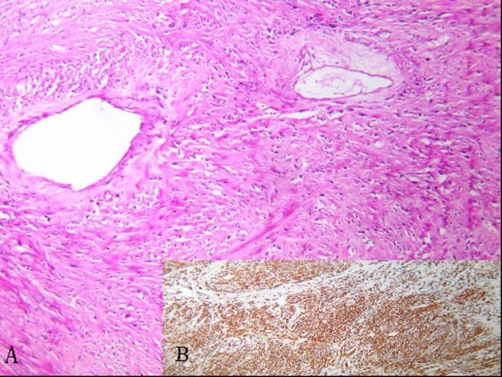
**(A)** Microscopic examination illustrating the monomorphic spindle cells (Hematoxylin & Eosin, 40×). **(B)** The immunohistochemical marking was smooth muscle actin +.

A 57-year-old woman African origin and Moroccan nationality was admitted with a history of a slowly enlarging, painless mass in the right supraclavicular region and shortness of breath on exertion. Upon physical examination, there was a hard right supraclavicular mass adherent to the deep structures of the neck. Uterine examination revealed normal. Biochemical and hematologic analyses were normal. Chest radiography displays a large opacity in the right apical thorax that displaced the trachea to the left side (Figure 1). The cervicothoracic Computed tomography scan demonstrated a solid heterogeneous cervical-mediastinal mass that displaced the trachea anterolaterally and compressed the oesophagus (Figure 2). The bronchofibroscopy revealed a tracheal extrinsic compression in the right reducing its lumen over 40%. There was no feeding artery of the mass in the arteriography. The abdomino-pelvic echographia is normal. On the basis of the clinical features, size, and radiographic appearance, we considered it like a benign and resectable tumor. A cervical collar incision was performed; the mass was adherent to the upper mediastinum and not related to the esophagus, trachea and superior vena cava. The tumor was dissected minutely and enucleated (Figure 3). The patient had an uneventful recovery and was discharged on the fifth postoperative day. Histologically, the lesion consisted of monomorphic spindle cells, no nuclear atypia or mitoses were observed, immunohistochemical stains revealed the tumor to be strongly positive for smooth muscle actin and desmin (Figure 4) and compatible with benign leiomyoma. Thirty month after surgery, the patient was still asymptomatic.

## Discussion

Leiomyomas mediastinal are extremely rare tumors which develop from smooth muscle, usually in the esophagus and large vessels (inferior vena cava, pulmonary artery, and superior vena cava). In our case, there was no tumor dependency to the neighboring structures. Although leiomyoma pathogenesis is obscure, estrogen and traumatic theories were suggested [2]. Primary mediastinal leiomyomas are frequently found at middle-aged women. Symptoms are rare and result mainly from the tumor local mass effect on vital structures. Baldo et al, reported a case of mediastinal leiomyoma presenting a superior vena cava syndrome [1]. Our patient had atypical location of this lesion; this is the second case of the leiomyoma mediastinal with cervical extension to be known. Computed tomography scan usually reveals the size and location of the tumor, however magnetic resonance imaging is more precise for demonstrating the extension of the lesion and its relationship with the adjacent structures [3]. Definitive diagnosis should be achieved by histology, microscopically; leiomyoma consists of monomorphic spindle cells with blunt-ended nuclei, arranged in interlacing fascicles. The specific immune marker, to smooth muscle actin, signs the diagnostic for leiomyoma.

Resection is the adequate treatment of primary mediastinal leiomyoma. If the tumor is vasculared, an embolisation should be performed prior to operating in order to control the bleeding during surgery. The local recurrence is uncommon, but if the mass recurs, the possibility of leiomyosarcoma should be explored [4].

## Conclusions

The primary mediastinal leiomyoma is exceptionally infrequent. It develops from small vessels in the soft tissue of the mediastinum wall. The tumor is surgically removed, and no local recurrence has been reported so far.
